# The relationship between effectiveness and costs measured by a risk-adjusted case-mix system: multicentre study of Catalonian population data bases

**DOI:** 10.1186/1471-2458-9-202

**Published:** 2009-06-25

**Authors:** Antoni Sicras-Mainar, Ruth Navarro-Artieda, Milagrosa Blanca-Tamayo, Soledad Velasco-Velasco, Esperanza Escribano-Herranz, Josep Ramon Llopart-López, Concepción Violan-Fors, Josep Maria Vilaseca-Llobet, Encarna Sánchez-Fontcuberta, Jaume Benavent-Areu, Ferran Flor-Serra, Alba Aguado-Jodar, Daniel Rodríguez-López, Alejandra Prados-Torres, Jose Estelrich-Bennasar

**Affiliations:** 1 Directorate of Planning, Badalona Serveis Assistencials SA, Badalona, Barcelona, Spain; 2 Medical Documentation, Hospital Universitari Germans Trias i Pujol, Badalona, Barcelona, Spain; 3 Psychiatry Department, Badalona Serveis Assistencials SA, Badalona, Barcelona, Spain; 4 Directorate of Planning, Badalona Serveis Assistencials SA, Badalona, Barcelona, Spain; 5 Jordi Gol i Gurina Primary Health Care Research Institute, IDIAP, Barcelona, Spain; 6 GESCLINIC – Corporació Sanitària Clínic, Barcelona, Spain; 7 Directorate of Primary Health Care, Consorci Sanitari Integral, Barcelona, Spain; 8 Health Sciences Institute of Aragon, Zaragoza, Spain; 9 Primary Health Care Management of Mallorca, Palma de Mallorca, Spain

## Abstract

**Background:**

The main objective of this study is to measure the relationship between morbidity, direct health care costs and the degree of clinical effectiveness (resolution) of health centres and health professionals by the retrospective application of Adjusted Clinical Groups in a Spanish population setting. The secondary objectives are to determine the factors determining inadequate correlations and the opinion of health professionals on these instruments.

**Methods/Design:**

We will carry out a multi-centre, retrospective study using patient records from 15 primary health care centres and population data bases. The main measurements will be: general variables (age and sex, centre, service [family medicine, paediatrics], and medical unit), dependent variables (mean number of visits, episodes and direct costs), co-morbidity (Johns Hopkins University Adjusted Clinical Groups Case-Mix System) and effectiveness.

The totality of centres/patients will be considered as the standard for comparison. The efficiency index for visits, tests (laboratory, radiology, others), referrals, pharmaceutical prescriptions and total will be calculated as the ratio: observed variables/variables expected by indirect standardization.

The model of cost/patient/year will differentiate fixed/semi-fixed (visits) costs of the variables for each patient attended/year (N = 350,000 inhabitants). The mean relative weights of the cost of care will be obtained. The effectiveness will be measured using a set of 50 indicators of process, efficiency and/or health results, and an adjusted synthetic index will be constructed (method: percentile 50).

The correlation between the efficiency (relative-weights) and synthetic (by centre and physician) indices will be established using the coefficient of determination. The opinion/degree of acceptance of physicians (N = 1,000) will be measured using a structured questionnaire including various dimensions. Statistical analysis: multiple regression analysis (procedure: enter), ANCOVA (method: Bonferroni's adjustment) and multilevel analysis will be carried out to correct models. The level of statistical significance will be p < 0.05.

## Background

The main factor determining the use of health services is the pattern of disease of the population, as shown by many studies in different geographic settings, care levels and health organization models [1]. In the hospital setting, nearly 20% of the variability in the use of health services is explained by the clinical characteristics of the population attended [2]. In primary health care (PHC), the explanatory power of the descriptive variables of the type of disease is still greater and may justify half of the variability in the use of resources, measured by frequency (visits or contacts), indirect consumption (referrals to specialists) and direct costs (diagnostic tests and analyses and drug prescriptions), rather than age, which is normally used to adjust resource distribution [3,4].

Patient classification systems (PCS) were introduced more than 20 years ago in order to measure patient characteristics. Of those developed for the hospital environment, Diagnosis Related Groups (DRG) are the most widely reported and used internationally [5]. DRG have been adopted by most public Spanish health administrations for planning and management for more than a decade [6]. However, in the PHC setting, these instruments are still in the research phase in many aspects, although they are beginning to be used in some regions (Aragon, Catalonia, Basque Country) as an aid to clinical decision making, health resource planning, economic distribution and epidemiological research, as they allow more reliable and accurate comparisons to be made between physicians than demographic population characteristics alone [7]. Of the different systems developed for PHC, Adjusted Clinical Groups (ACG) [8] is the most widely used.

However some factors still limit the widespread application of ACG. These include: 1) technical requirements (computerized medical records, the normalization and codification of medical terminology, the level of development of the classifications systems themselves and their validation in geographic and cultural settings and care models other than those for which they were created); 2) clinical interpretation of the patient categories identified by ACG and their potential use in clinical management and, 3) physician distrust of the application of tools aimed at objectively measuring and quantifying patterns of medical practice.

The technical requirements necessary for the application of ACG have improved substantially. Although not yet a complete reality, advances in computerizing medical records in PHC mean large databases in different geographic settings are now available. In addition, systems for automatic coding of diagnoses according to the International Classification of Diseases (ICD-9-MC) have been developed.

The team who designed and validated ACG [9] has developed new methodological approaches to categorizing the disease types that are closer to the global conception of the health status of PHC physicians and to the determining role that chronic disease may play in the use of services and its clinical management. In addition, predictive models of utilization have been designed that can identify population groups who are potentially very-high consumers of resources [10,11], classify the types of patients attended, and allow cost forecasts to be made. At present, these are the best-validated method of risk-adjustment in the Spanish health context.

ACG can be used for more precise and equitable financial decision-making and evaluate the efficiency of the use of health resources [12-14]. Currently, in Spain, some management groups promote the separation of financing, purchasing and provision of services, but require more accurate instruments to measure care activity. In recent years, there has been increasing interest in the use of financing per capita as a mechanism for the allocation of care resources [15-20].

Spanish health authorities are not convinced that PCS are able to respond to the management needs of PHC: there is still no normalized Minimum Basic Data Set (MBDS-PHC) in Spain and there seem to be doubts on the efficacy of PCS and their adjustment to the characteristics of a health context different to that for which they were designed [21].

Generally, the available evidence on the theoretical application of ACG is consistent, although there are few reports on practical application that reinforce this theoretical evidence. Various studies have measured the efficiency of health centres and physicians adjusted by the burden of morbidity, although these are case-mix indicators related to efficiency (cost-adjusted) and do not show whether the efficiency is associated with perceived or objective health outcomes. One criticism of PCS is that they do not allow the effectiveness or power of resolution of PHC teams to be determined, making them remote from daily clinical practice. In addition, there are currently no population-based studies that associate these aspects of efficiency and effectiveness in terms of management [22].

In Spain, the lack of large, reliable data bases makes this type of study difficult. Limitations in PHC computer systems include: a) measurement of total costs per PHC patient (fixed/semi-fixed and variable), and b) the provision of indicators for the evaluation of effectiveness (indicators of process, efficiency and evidence-based results) that measure the variability of clinical practice [11].

The justification of this project is that it will allow our team to build on their accumulated experience of PCS in the PHC setting by further refining their scientific consistency and extending it to unknown aspects such as the organisational behavior and physician profiles with respect to all resources available in PHC centres (laboratory, radiology, diagnostic/therapuetic tests, referrals and drug prescriptions).

We will attempt to establish and generalize the hypothesis that an improvement in the use of resources and direct costs adjusted by case-mix, would result in improvements in the clinical effectiveness of PHC teams. In addition, the acceptance of ACG by physicians is essential if clinical management indictors are to be included in the programming of contracts and could greatly aid decision-making in health management [23].

### Objectives

The main objective of this study is to measure the relationship between morbidity, the direct cost of health care and the degree of clinical effectiveness of selected Catalonian PHC centres and physicians by the retrospective application of ACG. The secondary objectives include: a) To determine the factors associated with a deficient correlation between the indices of effectiveness and efficiency (by PHC centre and physician): b) To determine the aspects conditioning the development and application of case-mix adjustment in PHC: c) To explore strategies for implanting ACG and, d) To determine the opinion of PHC physicians on the practical utility of case-mix adjustment systems.

## Methods/Design

### Study design and population

This is a retrospective multicentre study based on the computerized medical records of ambulatory patients and population data bases in Catalonia, a region in the northwest of Spain and a population of 7.5 million, within the framework of a larger Spanish study. The study population will consist of people of both sexes attending 15 PHC centres in Catalonia administered by five providers (Badalona Serveis Assistencials: Apenins-Montigalá, Morera-Pomar, Montgat-Tiana, Nova LLoreda [2] and ABS La Riera [2]; Consorci Sanitari Integral: Gaudí, Sagrada Familia, Torrassa and Collblanc; GesClínic: Les Corts; Consorci d'Atenció Primària de Salut de l'Eixample: Eixample and Rosselló; PROSS: La Roca del Vallés), with an assigned population of 350,000 of whom 15.1% are aged ≥ 65 years.

The population is mainly urban, with a lower-middle socioeconomic level and predominantly engaged in industry, commerce and services; organization of PHC teams is modernized, with public management and private provision of services concerted with the Catalan Health Service (Catsalut). Policies on staffing, training levels, organisation and services offered are representative of most PHC centres in Catalonia, with decentralized management and centralized structural services. All patients demanding care during 2008 will be included; patients transferring out to other centres, patients from other regions or countries and those attended by odontologists during the study period will be excluded. In addition, it is hoped to obtain information from 1,000 structured interviews with permanent staff (management, health and nonhealth).

### Main measures

Dependent variables will include the mean number of visits, care episodes and total direct costs of PHC care. Additional variables studied will include: a) general: age, sex, centre or team, basic activity unit (BAU) and number of visits carried out, b) total direct costs, c) comorbidity, d) variables of effectiveness and e) associated factors and user opinion. Patients will be assigned to medical specialties according to age: children aged 0–14 will be assigned to the pediatric service, and people aged > 14 years to the family medicine service. A visit will be defined as any contact between PHC teams and a patient in PHC centres or at home due to a demand for care or a health problem. If two physicians are involved in the same process, the activity will be ascribed to the physician of record.

Annual coverage (intensity of use) will be defined as the ratio: patients attended/assigned population. An episode will be considered as a process of care of a disease or an explicit patient demand (contact with health services) and will be quantified according to the International Classification of Primary Health (ICPC-2) [24].

Episodes occurring in the study population will be accounted for by the date of registration in the clinical history for each episode/reason for consultation, whether acute or chronic, regardless of the date of initiation of the diagnostic process. ICPC-2 will be converted (mapped) to ICD-9-MC by a five-strong working group (one information retrieval officer, two clinical physicians and two consulting technicians). The criteria followed will differ according to whether the relationship between the codes is null (one to none), univocal (one to one) or multiple (one to several).

### Case-mix measures

The ACG^® ^Grouper version 8 functional algorithm  is composed of a series of consecutive steps which result in 106 ACG, which are mutually-exclusive for each patient attended [8,9]. The construction of an ACG requires the age, sex and the reasons for consultation or diagnoses codified according to the ICD-9-MC.

The process of converting ICD-9-MC to ACG consists of 4 stages: the first two group a series of conditions according to similarity of resource consumption and the second two combine the most-common groupings: a) ICD-9-MC diagnoses are grouped into 32 Ambulatory Diagnostic Groups (ADG), of which a patient may have one or more; b) ADG are transformed into 12 Collapsed Ambulatory Diagnostic Groups (CADG); d) CADG are transformed into 25 Major Ambulatory Categories (MAC); and e) MAC are transformed into ACG.

This assigns each patient to a resource iso-consumption group. The method provides the USA mean relative weights (MRW) of each group with respect to the mean total cost, providing the resource utilization bands (RUB), which group each patient into one of five mutually-exclusive categories (1: healthy users, 2: low morbidity, 3: moderate morbidity, 4: high morbidity, and 5: very-high morbidity) according to morbidity, and the Expanded Diagnosis Clusters (EDC), which select patients by specific condition.

This tool allows specific episodes to be identified without the application of the algorithm of assignation of an ACG. The population attended will be considered as the standard (with identical criteria of inclusion and exclusion). The risk index (RI) reflects the complexity of the diagnoses attended by a centre with respect to the standard (case-mix).

The ratio: mean number of visits obtained using ACG/standard, will be calculated by indirect standardization. The efficiency index (EI) will be established as the ratio: number of visits/expected visits for all patients (indirect standardization). An RI or EI value equal to one signifies equal complexity or efficiency to the standard (year 2008), whereas an EI < 1 symbolizes greater efficiency (inverse relation). The relative cost of each ACG will be obtained by dividing the mean cost of each category by the mean cost of the reference population. This provides the MRW of each ACG group with respect to the mean total cost [8,9].

### Model of costs and use of resources

The design of the system of partial costs will be defined considering the characteristics of the PHC centre, the information requirements and the degree of development of the information systems available. The cost per patient attended during the study period will be taken as the unit that will serve as a basis for the final calculation.

The adaptation (conciliation or depuration) of expenditures from the profit and loss account of financial accounting to the costs of analytical accounting will be carried out in two stages: a) conversion of natural expenditures into costs and b) allocation and classification of costs. In the first stage, expenditures not directly related to the productive care process, i.e, financial expenditure, losses due to fixed assets, exceptional expenditure, stock variations and provisions for accounting years anterior and posterior to that studied, will be excluded. In addition, purchasing of intermediate products unrelated to radiology and laboratory or diagnostic tests will be excluded since these are considered variable costs or costs of productivity.

Natural costs will include the following concepts: staff (wages and salaries, indemnifications and social security paid by the company), consumer goods (medicaments, intermediate products, health material and instruments, using the formula; cost of purchases – remaining stocks), and expenditure on external services (cleaning, laundry), structure (building repair and conservation, clothes, office material), and management of the centre (write-downs and taxes). Expenditures will be periodified at the end of the financial year (accrued income): write-downs of fixed assets will be based on constant annual depreciation according to the useful life of the goods according to the Spanish General Accounting Plan for Health Centres; the cost of write-downs of capital goods will not be considered. In a second stage, costs will be assigned and classified.

The fixed or semi-fixed costs (imputation criterion: indirect costs) and variable costs (imputation criterion: direct costs) will be considered according to their dependence on the volume of activity carried out by the centres,. Direct costs will be considered as those related to physician diagnostic and therapeutic requests and referrals.

The study concepts included will be: laboratory (hematology, biochemistry, serology or microbiology tests requested; mean cost per request), conventional radiology (simple radiology, contrast radiology, ultrasound scans, mammograms and orthopantomography; tariff per test requested), medical transport (transfers by ambulance; mean cost per request), diagnostic tests (digestive endoscopy, electromyography, spirometry, teleradiography, computed tomography, audiometry, densitometry, ambulatory blood pressure, campimetry, stress tests, heart ultrasound, and others; tariff per test requested), interconsultations (urgent or regular referrals to reference specialists or hospitals; adapted tariff per reference), prescriptions (acute, chronic or on-demand prescriptions to Catsalut: retail price per package).

The tariffs used will come from the analytical accounting carried out by PHC centres, invoices of intermediate products issued by the different providers and the prices established by Catsalut. Natural expenditures (consumer goods, external structural and management services) will be considered as fixed or semi-fixed costs (imputation criterion: indirect costs). Various distribution alternatives to possible care or non-care cost centres will be evaluated by primary distribution to the family medicine and pediatric services of each centre. The mean cost per visit will be calculated and a final direct distribution for each patient will be made. Therefore, the cost per patient (Cp), according to the final service assigned will be: Cp = (mean cost per visit * number of visits [indirect costs]) + (variable costs [direct costs]). The cost model will not include the costs of specialized care (hospitalizations, emergency department, consultations, etc.) or social health (long stay, palliative, etc.)

### Measure of effectiveness

Effectiveness will be defined according to a set of indicators of process and results (differentiated for family medicine and pediatric services). A general synthetic index (SI) will be constituted from the selected indicators. Each indicator will be specifically defined and calculated. The SI will be calculated by ajdusted rescaling with percentiles (median; percentile 50) and will be drawn up according to population-based indicators, chronic disease processes, health outcomes, efficiency, quality of prescriptions, vaccines for risk groups and user complaints. Thirty indicators for family medicine and 20 for pediatrics will be established.

### Final procedure

The adjusted EI of visits, referrals, drug prescriptions and the total EI will be established, enabling physician profiles to be drawn up. Posteriorly, the EI and SI will be correlated for each centre and BAU. Subsequently, factors associated with PHC centres and physicians with inadequate indices of efficiency and effectiveness will be quantified and structured in accordance with the characteristics of the centres and professional profile.

Physician opinion will be measured using a structured, anonymous questionnaire containing closed questions on the dimensions of knowledge, applicability and satisfaction with ACG in clinical practice.

### Power calculation to determine sample size

With an expected synthetic index of 50% and assuming a random error of 5% (95% confidence interval), a normal distribution (bilateral contrast) and an estimated precision of 2‰, the sample size to be recruited will be 240,092 patients. The statistical power of the model will be 80%.

### Statistical analysis

Before the statistical analysis, all data, and especially the medical histories (OMIAP-WIN programme), will be carefully reviewed, observing the distributions of frequency and searching for possible errors in recording or codification. The data will be obtained in computerized form and the confidentiality of records will be respected at all times according to Spanish law (LO del 15/1999 de 13 de diciembre, de Protección de Datos de Carácter Personal, LOPD).

The study variables will be evalauated using the Kolmogorov-Smirnov conformity test. Dependant variables (direct episodes, visits and costs) will be transformed using the Naperian logarithm. Depuration of these variables will be made to establish the cut-off point (T) of extreme cases (atypical observations) using the formula: T = Q_3_+1,5 (Q_3_-Q_1_), where Q_3 _and Q_1 _are the third and first quartiles of the distribution, respectively. The explanatory power of the classification will be calculated using the ratio: determination of intra-group variance/total. The homogeneity of variance will be tested using Cochran's test. The association between quantitative variables will be evaluated using Pearson's linear correlation and/or Spearman's rank correlation coefficient. Multiple linear regression analysis (procedure: enter) and covariance (ANCOVA; procedure: Bonferroni) will be carried out according to the recommendations of Tompson and Barber for the correction of the models used. In addition, a multilevel, analysis, where the variables, centre and physician, will act as components, will be made. The level of statistical significance will be established as p = .05. The analysis will be made using the SPSS v12 for Windows programme.

### Approval by the Committee of Ethics and Clinical Research

In accordance with Spanish recommendations, the Study Protocol was approved by the Committee of Ethics of Clinical Research of the Foundation Gol and Gurina (IDIAP).

### Study timeline

#### 1) Year One

Phase 1–2. Meeting to decide general planning of the study.

Tasks will be assigned to investigators and informative meetings held with physicians from particpating PHC centres. Posterior followups will be quarterly. A bibliographical and documental search will be made and a structured summary drawn up.

Phase 3. Obtention of patient variables (quantitative information). Time: 8 months.

This includes: a) design and drawing up of a morbidity data base (care episodes attended by patient/year). Time: 1 month; b) design and drawing up pharmaceutical prescription data base (Catsalut). Time: 2 months; c) design and drawing up direct costs data base (laboratory, radiology, referrals and pharmaceutical prescription per patient/year). Time: 3 months; d) conversion (mapping) of CIAP to ICD-9-MC. Time: 1 month; and e) obtention of an ACG per patient/year. Time: 1 month.

#### 2) Second year

- Phase 4. Drawing up of a computer programme for depuration of dependant variables, using MS Access and Visual Basic. Time: 1 month.

Phase 5. Drawing up of data base of 50 indicators of effectiveness of clinical practice. Previous bibliographical search based on evidence available. Time: 2 months.

Phase 6. Unification of quantitative and qualitative data bases. Time: 1 month.

#### 3) Third year

Phase 7. Design and carrying out of interviews with physicians. Time: 5 months.

Phase 8. Validation of data quality. Verification of univariate results by ranges. Identification of inadequate categories. Time: 2 months.

Phase 9. Data analysis. Including: a) statistical analysis: descriptive, bivariate and multivariate; b) analysis of interviews: structured questionnaires; and, c) interpretation of the results. Time: 5 months.

Phase 10. Scientific diffusion of the results: this will include the writing, translation and publication of the results obtained. Time: One year.

### Data confidentiality

Confidentiality was respected at all times according to Spanish law (LO del 15/1999 de 13 de diciembre, de Protección de Datos de Carácter Personal, LOPD).

## Discussion

We expect the greatest limitations of the study to be related to the degree of development of maturity of information systems, the accuracy of conversion of ICPC-2 to ICD-9-MC, the accuracy of the measurement of costs and the efficiency or possible variability and/or severity in the selection of the care episode by physicians, which might lead to contamination between groups or a lack of clinical specificity. However, these unknown factors may be minimized by the large study population we intend to recruit (regression to the mean). It is expected that the results will have significant practical applications. The practical characteristics of ACG mean they could become one of the most-important risk-adjustment intruments in Spain in the future with respect to the financing of health centres. It is also expected that the study will have significant applications in clinical practise.

Firstly, a PCS is used and EI obtained in the visits and the use of diagnostic tests, referrals and pharmaceutical prescriptions provides a professional profile. Secondly, knowledge of these EI and their relationship with health outcomes (SI, effectiveness) is important, not only for clinical management, but also to allow health professionals to learn how well they are working. It is hoped that the study may permit changes in organisational models with respect to the optimization of resources (healthy children, follow-up of chronic diseases, etc.) and improvements in obtaining information (real time, creation of a corporative web of the results) and a follow-up of the health outcomes for physicians. Continuous improvement in these aspects may result in improvements in overall quality, with repurcussions in the control of patients. In addition, according to the results observed, it is hoped that these indicators may gradually be included in contracts for health professionals including incentive programmes. The results will be shared with participating centres by means of clinical sessions and meetings with management.

## List of Abbreviations used

ACG: Adjusted Clinical Groups; ADG: Ambulatory Diagnostic Groups; PHC: Primary Health Care; RUB: Resource Utilization Band; CADG: Collapsed Ambulatory Diagnostic Groups; ICPC-2: International Classification of Primary Health Care; ICD 9-MC: International Classification of Diseases; MBDS-PHC: Minimum Basic Data Set-Primary Health Care; EDC: Expanded Diagnostic Clusters; DRG: Diagnosis Related Groups; EI: Efficiency Index; SI: Synthetic Index; MAC: Major Ambulatory Categories; MRW: Mean Relative Weight; PCS: Patient Classification System; NHS: National Health System; BAU: Basic Activity Unit

## Competing interests

Research project financed by Fondo de Investigaciones Sanitarias de la Seguridad Social (Instituto Carlos III, Majahonda (Madrid), Spain; Reference: PI 08/1567).

## Authors' contributions

AS, RN, MB, JV, JB, AA, DR and SP drew up the study protocol and structured the bibliographical search. AS, JV, AA, CV and EE will carry out data obtention. RN, MB, BS, EE, JL, JV, JB, NS, FF, AA, DR, SP, JE and SP will carry out the analysis and interpretation of the initial results. RN, MB, AA, NS, BS, JV and CV will draw up the efficiency indicators. BS, EE, NS and AS will structure the questionnaire for health professionals. All authors will contribute ideas, interpret the findings and review rough drafts of the manuscript. All authors will approve the final versions of all manuscripts. AS is the head of the Catalan study.

## Pre-publication history

The pre-publication history for this paper can be accessed here:


